# Raman Scattering Analysis of High Explosives on Human Hair: From Aromatic (TNT) to Aliphatic (RDX and PETN)

**DOI:** 10.3390/ijms26209913

**Published:** 2025-10-12

**Authors:** Francheska M. Colón-González, María A. Villarreal-Blanco, María P. García-Tovar, Priscilla D. Soler-Rodriguez, Tatiana P. Serrano-Zayas, Giancarlo L. Vargas-Alers, Emanuel Ocasio-Reyes, Luis. A. García-Cruz, John R. Castro-Suárez, Nataly J. Galán-Freyle, Leonardo C. Pacheco-Londoño, José A. Centeno-Ortiz, Samuel P. Hernández-Rivera

**Affiliations:** 1 Department of Chemistry, University of Puerto Rico-Mayagüez Campus, Mayaguez, PR 00681-9000, USA; maria.villarreal@upr.edu (M.A.V.-B.); maria.garcia87@upr.edu (M.P.G.-T.); priscilla.soler@upr.edu (P.D.S.-R.); tatiana.serrano1@upr.edu (T.P.S.-Z.); giancarlo.vargas@upr.edu (G.L.V.-A.); emanuel.ocasio4@upr.edu (E.O.-R.); luis.garcia48@upr.edu (L.A.G.-C.); jose.ortiz10@upr.edu (J.A.C.-O.); 2 Exact Basic Area, Universidad del Sinú, Unisinú, Cartagena 130015, Colombia; johncastrosuarez@gmail.com; 3 Life Science Research Center, Universidad Simón Bolívar, Barranquilla 080002, Colombia; nataly.galan@unisimon.edu.co (N.J.G.-F.); leonardo.pacheco@unisimon.edu.co (L.C.P.-L.)

**Keywords:** High Explosives (HEs), human hair, Raman Scattering (RS), Multivariate Analysis (MVA), Principal Component Analysis (PCA), Forensic Sciences (FS)

## Abstract

There is a need to develop rapid, in situ methods that require less sample preparation and lower limits of detection for the detection of High Explosives (HEs). Considering that human hair is one of the primary attributes of the human body, its presence can be used to identify possible traces of hair evidence for forensic screenings. Using non-invasive in situ approaches coupled with multivariate analysis (MVA) can enable rapid detection, thereby decreasing analysis time and reducing the cognitive load on analysts, with response times as low as milliseconds or lower. This preliminary study demonstrates the detection of 2,4,6-trinitrotoluene (TNT), 1,3,5-trinitroperhydro-1,3,5-triazine (RDX), and pentaerythritol tetranitrate (PETN) on black, bleached, and natural gray human hair coupled with principal component analysis (PCA). It was possible to discriminate the HE signals from those of the substrates (hair types) on black, gray, and bleached hair by monitoring characteristic peaks for the nitro group’s vibrations of the explosives. Gray hair presented good discrimination for the explosives due to the absence of melanin. The best modes for discriminating HEs from all three hair types were identified using PCA, with data pretreatment based on the first and second derivatives of the algorithms. The classifications were based on the more substantial variation in the NO_2_ symmetric vibration for each HE.

## 1. Introduction

The detection of high explosives (HEs) is an issue of high impact at the security level since, for years, it has sought to combat global terrorism. Various explosives, such as TNT, RDX, PETN, DNT, TATP, etc., have been detected with vibrational techniques in substrates such as stainless steel [1], soil [2], aluminum, fabrics, and others [3]. HEs on hair have been detected by different chromatographic methods [4,5]. Although these techniques are very sensitive and selective, they are expensive techniques; more complex sample preparation is required, and solvent extraction techniques must be used to extract the HEs in hair, which indicates that it can be a laborious and time-consuming process [2,6]. The best HE selectivity could be possible using spectroscopic methods, yielding a fingerprint with a wealth of information that permits almost certain identification [7]. However, univariate analysis, which uses just one or two of the HEs’ distinctive peaks, has been a preferred analysis methodology for many years. According to the complexity of spectral data, models are needed to categorize and measure the target analyte.

Due to the complexity found in spectral data, models to classify and quantify the desired analyte are required. For years, screening has been performed using univariable analysis, in which only one or two characteristic peaks of the HEs are used for detection. Previous studies have also addressed the detection of HEs by using spectroscopy and chromatographic methodologies [1,2,3,4,5,8,9,10]. This methodology can be improved using a chemometric approach, which is entirely multivariable [11,12,13]. Multivariate analysis (MVA) helps find structure amongst variation, in this case of the spectral data, and establishes patterns or behavior in the data. Based on previous results from our research group and literature, chemometrics in MVA has been effective for identifying, quantifying, and discriminating threats among interferences in Raman Scattering (RS) data. When the spectral data has been acquired qualitatively, Principal Component Analysis (PCA), an exploratory MVA model [14,15], is usually applied. PCA is a linear dimensionality reduction technique that transforms spectral data into a new coordinate system to highlight variance and underlying patterns [16]. It decomposes the data into scores, loadings, and residuals, which together help identify chemical variation, detect outliers, and classify unknowns [16,17]. PCA has proven effective in exploring and identifying chemical signatures, including HEs, in spectroscopic datasets [18].

Previous studies have demonstrated the potential of RS combined with chemometric tools for the detection and differentiation of explosives on diverse substrates. For instance, RS detection of TNT, RDX, and PETN has been reported on metals, soils, fabrics, and reflective surfaces, showing characteristic nitro group vibrations that allow classification. [19] Similarly, PCA has been successfully applied to discriminate between aromatic and aliphatic explosives by enhancing spectral resolution and clustering patterns [20]. Building on this literature, the present work extends RS/PCA approaches to human hair substrates, which represent a complex biological matrix where fluorescence and pigmentation introduce new challenges for classification. Unlike previous work that relied on single peak analysis, our approach uses multivariate PCA to capture the full spectral variability and improve discrimination across hair types.

In this research, the primary objective is the qualitative discrimination of HEs on human hair. To achieve this, PCA was applied to the RS spectra to improve visualization of clustering patterns and to support the identification of HE signatures. The results will contribute to the development of a spectral library that can be used for future reference by researchers and government agencies. The following article will discuss the PCA for the raw data, how this data is then reprocessed to obtain a better visualization of the data, the verification of why the data is behaving the way it is, and finally, corroboration of these details by using the loading plots compared with the respective contribution of the HE.

## 2. Results

**Figure 1:**
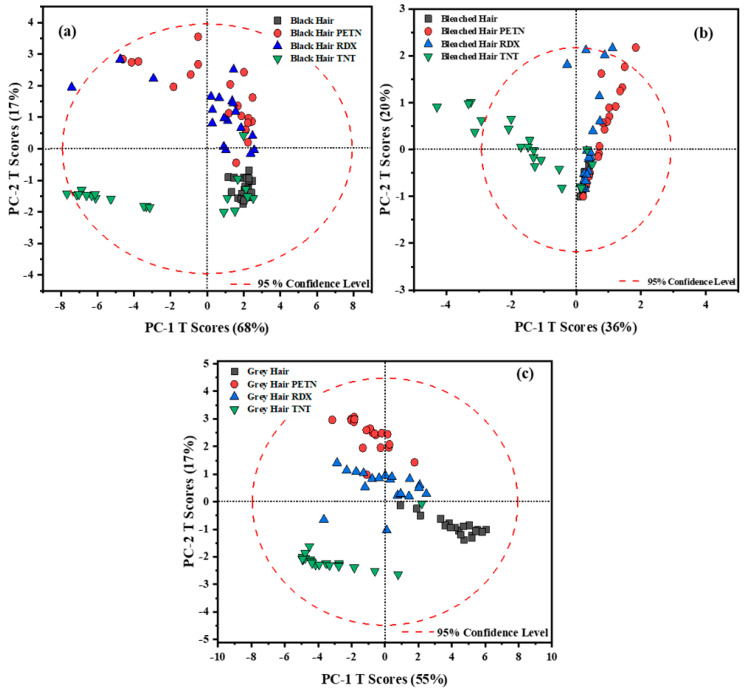
Scores plot obtained for the raw data for hair samples with HEs for (**a**) black hair, (**b**) bleached hair, and (**c**) gray hair.

PCA was used as an exploratory tool to qualitatively identify possible information between traces of explosives on human hair. After the spectral data were characterized, PCA models for black, bleached, and gray hair were generated. Score plots based on PCA models were developed. The first score plot for each type of hair sample is shown in Figure 1. For all three score plots, the principal components PC-1 and PC-2 showed the most significant variations in the spectral data. For black hair, the two PCs contained 85% of the variance. For the other hair types, the variance captured by the PCs was 56% for bleached hair samples and 72% for gray hair samples (Figure 1). Even though black hair presented the most substantial contribution to the total variance on the first PCs, gray hair exhibited a better separation between samples per HE. Each of the PCA models effectively separated the TNT samples into clusters, but it still lacked the ideal separation for specific samples whose spectral vibrations may not be as well-defined. This is due to the differences between the -NO_2_ vibration and the vibrations of the substrates. PETN and RDX are aliphatic HEs, and TNT is a nitroaromatic HE. These chemical variations can explain the behavior of the score plot for black and bleached hair types. Another reason attributed to the separation between HEs is the effect of fluorescence on the spectra. An interesting aspect of obtaining a PCA score plot is evaluating the variability between each sample (in this case, the HE) and how the model behaves depending on their characteristic vibrational peaks. Figure 1 presents the PCA score plots for the raw data, illustrating that the separation and distribution of the data do not allow the model to easily distinguish between RDX and PETN across the different hair samples (Score plots a–c).

**Figure 2:**
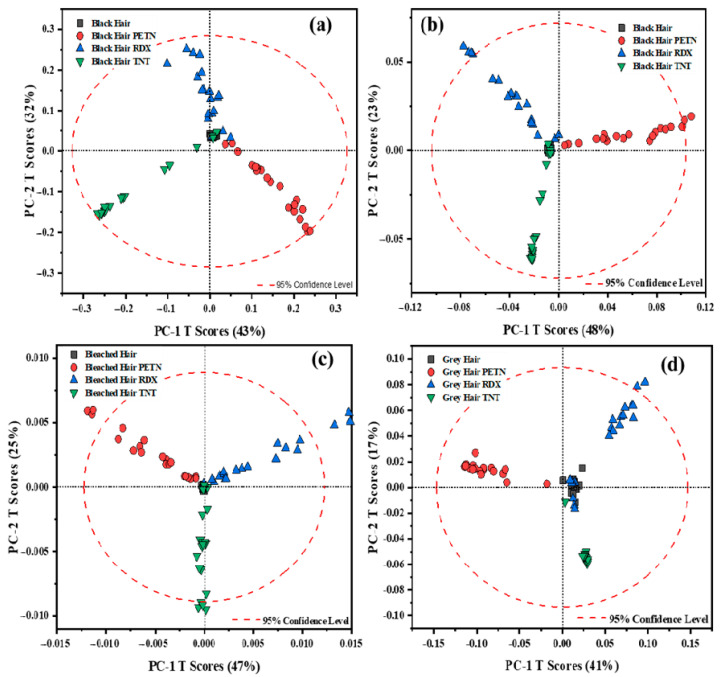
Plots for data treated for first and second Savitzky–Golay derivatives: (**a**) black hair pre-processed data with the first derivative; (**b**) data pre-processed with the second derivative. The second derivative is applied to the spectral data for (**c**) bleached and (**d**) gray hair.

For this reason, pretreatments were performed on the data. The best pretreatment for the spectral data resulted in the Savitzky–Golay first and second derivatives. Derivatives are the most common treatments applied to spectral data to resolve peak overlapping (thus enhancing spectral resolution) and to eliminate constant and linear baseline drifts between samples [21]. One disadvantage is that the application of derivatives could increase noise, but this can be evaluated using the loadings to determine which variables affect the variation in the data. For the pre-treated data, derivatives and a 2nd order polynomial fit of 15 pts. were used to obtain the score plots shown in Figure 2. For these plots, better data visualization was achieved for each hair sample. Other pretreatments were analyzed: multiplicative scatter correction (MSC) and standard normal variate (SNV), which both work for scatter correction. SNV specifically reduces the spectral data’s scattering, particle size, and intensity [22]. The data was adjusted in the MSC to match the reference spectrum’s amount of scattering. Before choosing the optimal model for the data, their combinations were constructed (such as inverting the order of application of the pre-treatments).

When analyzing the data, we considered two key factors: first, the variance explained by the first two PCs, and second, the clarity of pattern separation among the samples. In the case of black hair, when comparing the variance explained, the model’s performance shifted from 85% with raw data to 75% using the first derivative, and further to 71% with the second derivative. Despite the slight reduction in explained variance, both pre-processing methods yielded superior clustering and separation of the HEs within the first two principal components, thereby improving the interpretability and discriminatory power of the model, enhancing the model’s ability to discriminate between the HEs across the hair samples. Although the first derivative was initially considered the most effective pre-processing method for black hair, given that the data clustered within the 95% confidence interval and highlighted significant variations in the -NO_2_ peaks for each HE, the second derivative provided better overall spectral separation across the remaining hair samples. Therefore, to maintain consistency in data analysis, the second derivative was ultimately adopted. In contrast, the spectral behavior of bleached hair followed a different pattern. The variance explained by the PCA score plot increased from 56% with raw data to 72% after applying the second derivative, enabling the model to capture more meaningful information within the first two principal components. This pre-processing step also led to improved clustering of the HEs. As illustrated in Figure 1b, although the model attempts to group the data, it also struggles to distinguish between RDX and PETN clearly. This limitation is likely also due to the elevated fluorescence background associated with bleached hair, which obscures the vibrational peaks critical for HE identification even further. However, as shown in Figure 2c, this issue is effectively mitigated, resulting in distinct separation among all three HEs. This suggests that the model is now clustering the data based on the dominant HE spectral features, thereby enhancing classification accuracy. In the case of both bleached and gray hair, the application of the first derivative failed to yield satisfactory model performance, as it did not adequately capture the underlying spectral features, as with other applied data pre-treatments. For gray hair, the score plot with a second derivative demonstrated that the variation changed from 72% to 58%, and the data were grouped better into clusters between each of the HEs. Gray hair data resulted in a better grouping between the HEs because of the lack of pigmentation, as seen on the raw score plot, Figure 1c. When analyzing the RS spectra for gray hair, it can be seen that the spectra are less influenced by fluorescence from the indole groups of melanin, resulting in better visualization of the data on the score plots (raw score plot and with the second derivative pretreatment) for this type of hair. Finally, for black and gray hair, although the variance captured by the first two PCs decreased slightly after applying first (only for black hair) and second derivative (for bleached and black hair) pretreatments, these transformations enhanced the visual distinction between sample clusters in the score plots. This suggests that the model, despite explaining slightly less variance, offers improved pattern recognition. Conversely, for bleached hair, the derivative pretreatment not only increased the variance explained by the first two PCs but also enhanced the separation of sample patterns. Therefore, this balances both statistical variance and visual clarity, which provides a more comprehensive understanding of model performance across different hair types.

In each score plot, hair samples without HEs were in the center, and the principal variation was attributed to the vibrational signals for each HE. The second derivative score plot clusters determine the similarity between the vibrations of each molecule, and this is why PETN, RDX, and TNT deposited on hair are fully grouped in different directions on the score plots. It should be mentioned that the entire spectral range was analyzed for these PCA models. Looking at the variation in the loadings plot, removing points that do not contribute to the interpretation (in the Raman Shift) is possible. Still, a complete analysis of the data is desirable. Points that do not contribute to the model can be removed since they do not have variation; this could improve the explanation percentage of the model’s data in fewer PCs.

**Figure 3:**
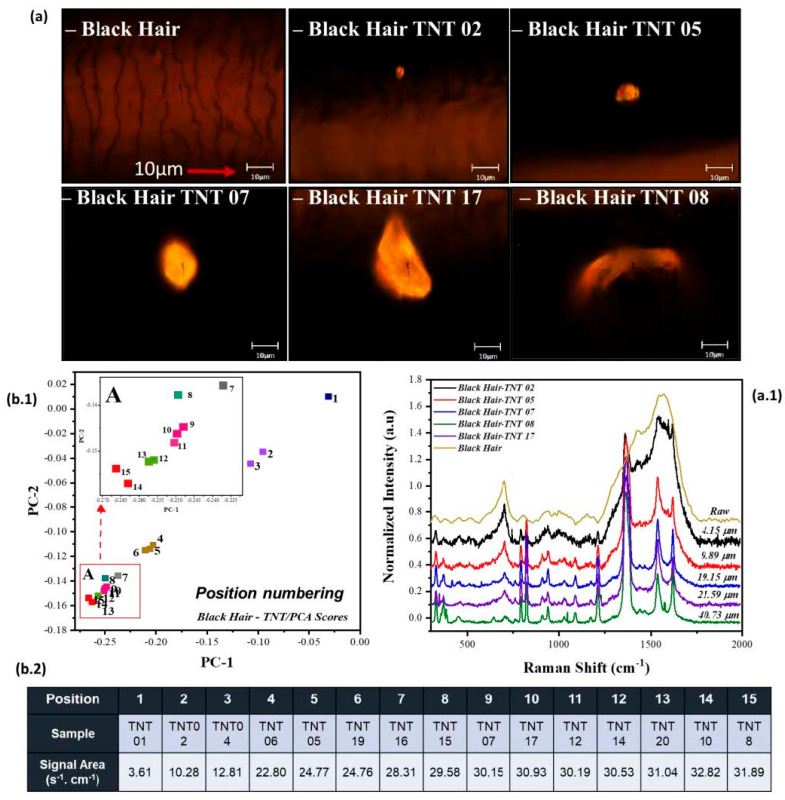
Distribution of Black Hair with TNT deposition. (**a**) Gray light micrograph of strands of black hair, along with (**a.1**) RS spectra showing the approximate diameter of each TNT crystal. In addition to the prominent -NO_2_ peak at 1369 cm^−1^, the spectral region between 750 and 1000 cm^−1^ shows intensity variations corresponding to additional TNT markers. These bands contribute to the chemical fingerprint used for explosive identification and are relevant for PCA-based clustering. (**b.1**) Score plot for black hair pre-processed with the first derivative, annotated using positional numbering radiating from the center. (**b.2**) Table emphasizing the grouping and direction of TNT samples with their respective signal area from the primary nitro group vibration (1369 cm^−1^) on the HE.

As previously mentioned, and shown in Figure 2, the variation for each HE is distributed in different directions, forming line clusters for each HE. A spectral trend within the line clusters can be identified when evaluating the variation in TNT’s prominent characteristic peak of -NO_2_, as seen in Figure 3. This variation is due to multiple factors. The areas of TNT’s -NO_2_ vibrational peak (centered about 1369 cm^−1^) were analyzed to prove one of these factors. The analysis shows a correlation between the increase in TNT’s -NO_2_ vibrational peak intensities and the distribution from the center and out of the score plot. The area of these vibrational peaks was acquired by using the Origin Pro™ 2020 software. Still, an approximation can be obtained with the area of a trapezoid. The area under the vibrational peak of -NO_2_ helps identify the important vibrational marker, and it can be interpreted as the approximate analyte (the HE) present in each sample. This information helps to understand how the model behaves with the added data. When analyzing the area under the vibrational peak of -NO_2_ for the RS of Sample 1, it can be observed that it has a 3.61 cm^−1^∙s^−1^ area. In contrast, the point located at position fifteen is from Sample 8 and has an area of 31.89 cm^−1^∙s^−1^ (from the normalized plot of the RS spectra). This means we can interpret this variation in the score plots due to the -NO2 vibration within the TNT samples and the variation on the line clusters for RDX and PETN.

Another relationship that was identified is the dependence of the integrated RS signal at 1369 cm^−1^ and the approximate “diameter” (or characteristic length) from each crystal deposited on the strand of hair, as seen in Figure 3a. Figure 3(b.2) illustrates the grouping of samples based on their spatial distribution radiating outward from the center of the PCA score plot. The term “sample” refers to the original identifier assigned to each hair specimen during collection and analysis. In contrast, “position” denotes the relative placement of each sample within the score plot, reflecting its distance and orientation from the central point of the PCA projection. Figure 3(b.1) illustrates the score plot distribution in line cluster for Black Hair-TNT samples, indicating the spatial arrangement of each sample radiating outward from the center of the PCA score plot. The approximate diameter of each crystal was acquired by approximating pixels on the image and converting them to micrometers using the scale on each of the gray light micrographs. The diameter of the laser spot was approximately 1.1 μm (50× Ultra-Long Working Distance, ULWD) Olympus™ objective (Olympus Corporation, Tokyo, Japan). (see Table 1: Predicted vs. Measured laser beam diameters for several ULWD Olympus™ ultra-long working distance microscope objectives). A notable relationship observed is that as the diameter of the HE crystal decreases, its spectral contribution to the RS signal diminishes, and vice versa. If the equipment is fully calibrated and the image is focused, it should follow this trend. However, in certain hair coloration types, the spectral signal from the HE crystal can be obscured by a strong fluorescence background. It is important to clarify that this fluorescence originates from the indole groups in the hair strand itself, not from the HE crystal. Therefore, the spatial positioning of the HE on the hair strand plays a critical role in evaluating this trend. When the HE crystal is located on the outer surface of the strand, its contribution can be reduced, allowing for the collection of more information from the hair strand (more fluorescence). On the other hand, if the HE crystal is localized in the center of the hair strand, when analyzed, it allows a more precise visualization of characteristic peaks such as the nitro group signal around 1369 cm^−1^ in the RS spectra.

**Figure 4:**
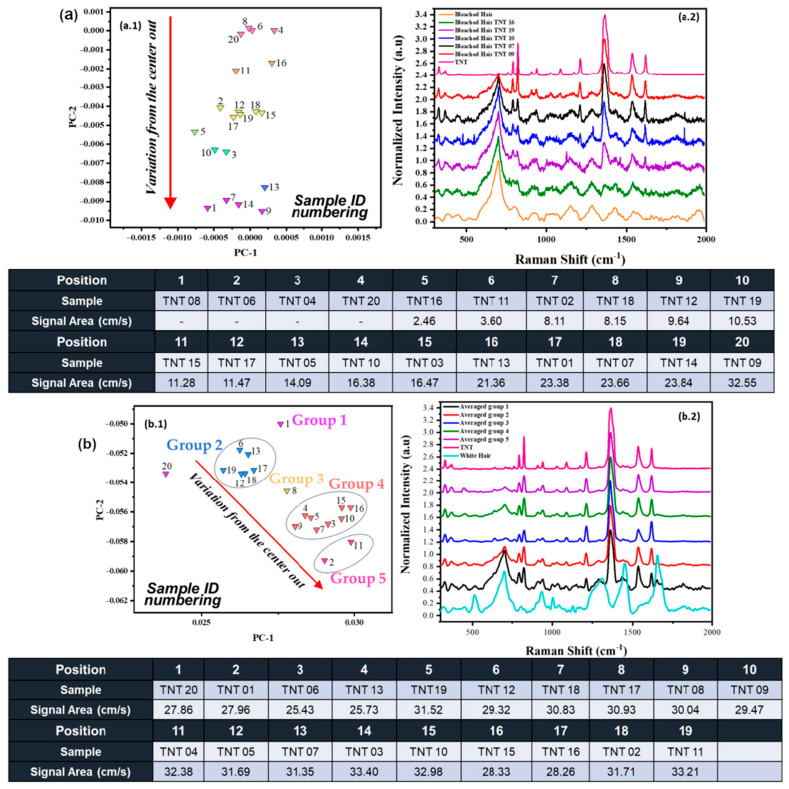
(**a**) Distribution from bleached hair with TNT deposition. (**a.1**) Score plot for bleached hair with the second derivative. Emphasis on grouping and direction of TNT samples with their respective areas from the primary nitro group vibration (1369 cm^−1^) on the HE. The numbering reflects the unique identifiers assigned to each sample (Sample ID). (**a.2**) RS spectra of different regions on the score plot for bleached hair + TNT. (**b**) Distribution of gray hair with TNT deposition. (**b.1**) Score plot for gray hair with the second derivative. Emphasis on grouping and direction of TNT samples with their respective area from the primary nitro group vibration (1369 cm^−1^) on the HE. The numbering reflects the unique identifiers assigned to each sample (Sample ID) (**b.2**) Averaged RS spectra for gray hair + TNT.

The distribution on the score plot was also analyzed for bleached hair samples, observing the same trend as for black hair, as seen in Figure 4a. Based on the evaluation of multiple RS spectra from bleached hair, as shown in Figure 4(a.2), we observed that the variance radiating outward from the center of the score plot is closely associated with the intensity of the nitro group peaks, as also shown in the black hair variations. This relationship contributes to the formation of line cluster distributions across the score plots for each hair sample. Unlike Figure 3(b.1), which uses positional numbering radiating from the center of the score plot, Figure 4(a.1,b.1) display sample identifiers (“Sample ID numbering”) directly on the score plots. It is important to note that this approach was chosen because multiple samples were located within overlapping areas, making consistent positional numbering impractical. The distribution depicted in the score plot is mainly due to the nitro group’s main peak on TNT (1369 cm^−1^), which will be explained in more detail later in the discussion. The evaluation was made for TNT, but the same variation occurred with aliphatic explosives, PETN, and RDX. For samples 8, 6, 4, and 20 for bleached hair-TNT, it was impossible to indicate the area from the -NO_2_ vibration (using the Origin Pro™ 2020 software), but still, the model recognized that those samples belong to this cluster. The diameter of the TNT crystals for samples 8, 6, 4, and 20 for bleached hair was not acquired due to small crystals, and the -NO_2_ vibration peak was masked by the bleached hair vibrational contribution. This is why it is efficient to use MVA methods since they detect even the most minor changes/variations in the spectral data.

For gray hair, the line clustering trend was not as accurate as for black hair and bleached hair, as the peak areas from the nitro group varied across different parts of the cluster. Various groups can be identified within the line cluster, illustrated as groups 1 to 5, as shown in Figure 4(b.1). Notably, some samples, such as 6, 13, 19, 12, 18, and 17, are in the same area of the cluster, but they present different variation areas (cm^−1^∙s^−1^) from each of their -NO_2_ vibration peaks at 1369 cm^−1^. When these groups were averaged, the trend of increment for the -NO_2_ band was followed similarly to the other hair coloration types, as seen in Figure 4(b.2). This grouping trend may be attributed to the fact that gray hair signals do not significantly interfere with the NO_2_ peaks due to the lack of pigmentation, allowing vibrational peaks from the explosives to be more clearly visualized across the RS spectra. For this model, not only are the vibrational features of the explosive being discriminated against, but also those inherent to gray hair. Since the explosive signal in gray hair is not as overshadowed by fluorescence, the model incorporates a broader range of spectral features beyond the nitro group peak. This results in slight positional variation for each sample within the score plot, reflecting the influence of these additional spectral contributions.

**Figure 5:**
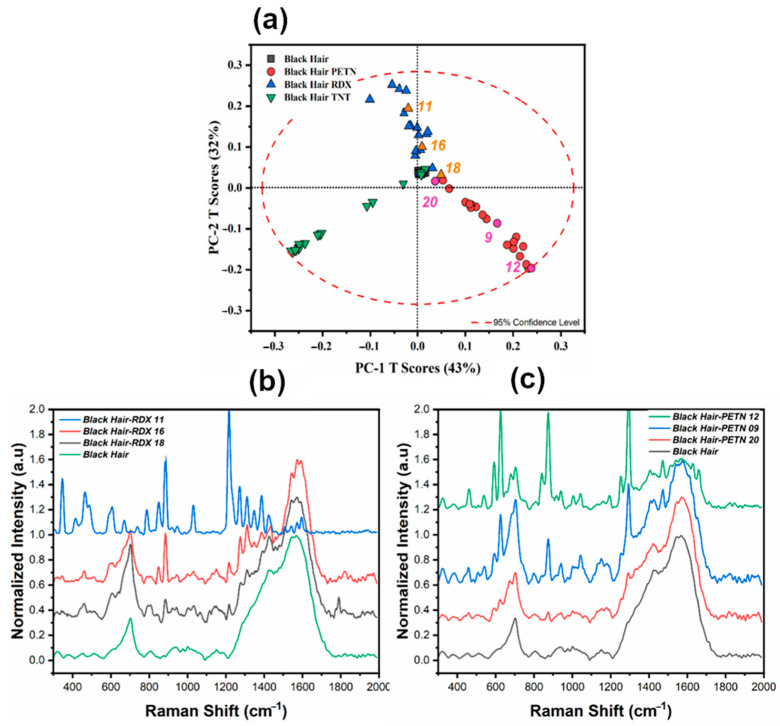
Distribution of Black Hair with RDX and TNT deposition. (**a**) Score plot for black hair pre-processed with the first derivative, with labeling (**b**) RS spectra comparison for score plot variation for RDX. (**c**) RS spectra comparison for score plot variation for PETN.

An in-depth analysis was conducted to examine the observed variation in the Black Hair samples, as illustrated in Figure 5, to observe whether the variation within the score plots for each HE was consistent. The distribution in the score plot consistently reflects this, indicating that the variation radiates outward from the center based on the contribution of the HE crystal in each sample, as depicted in Figure 5b,c.

**Figure 6:**
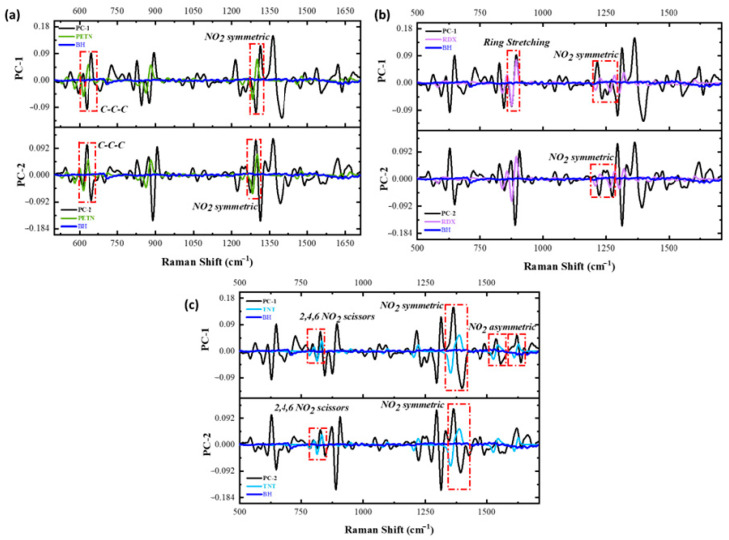
(**a**) Comparison between the first derivative of the calculated PC-1 (upper part of the graph; black trace) and PC-2 (lower part of the graph; black trace) of the Loading Plot (**a**) for black hair/PETN and first derivative for a PETN and black hair reference RS spectrum (green and blue trace, respectively); (**b**) for black hair/RDX and the first derivative for an RDX and black hair reference RS spectrum (magenta and blue trace, respectively); and (**c**) for black hair/TNT and the first derivative for a TNT and black hair reference RS spectrum (aqua and blue trace, respectively).

**Figure 7:**
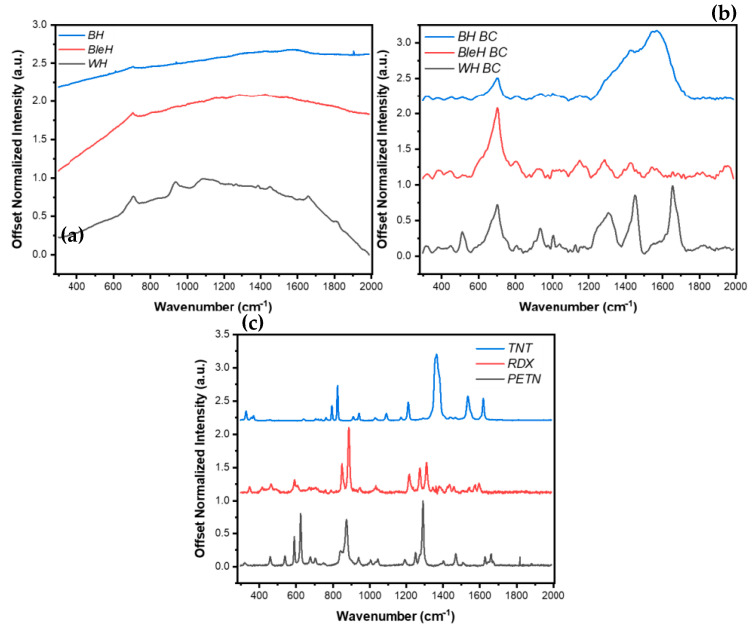
Corresponding RS spectra for hair samples (**a**) before and (**b**) after baseline correction (BC) in the spectral range of 200cm^−1^ to 2000cm^−1^. (**c**) RS spectra of HE (TNT, RDX, PETN) in the spectral range of 200cm^−1^ to 2000cm^−1^. The raw spectra illustrate the fluorescence background and characteristic vibrational peaks of each hair sample, while the corrected spectra highlight the distinct signature peaks used for PCA-based classification. This comparison demonstrates the effectiveness of baseline correction in enhancing spectral clarity and facilitating discrimination between explosive types on different hair substrates.

The vibrational signals from each HE can be evaluated using the obtained loading plots to fully corroborate that the variation being analyzed is the one from each of the vibrations of the nitro groups. The RS spectral region from 311 to 1750 cm^−1^ was used to investigate these possible variations. This spectral region was selected because most of the variation for the model was situated here. The loading plot obtained for the PCA model for black hair was evaluated to identify variation in areas from the spectral data. The loadings presented are from the pre-treated data using the first derivative. Figure 6 represents the loadings plot variation for the first two PCs of the model in comparison with each of the HEs of interest. When a first derivative is applied, each of the vibrations is located at the intersection of the x-axis. The main variations occur in the vibrational signals from each of the explosives. The results from these vibrations are presented in Table 2, and Figure 7 provides the spectra for each of the HEs under study and each of the hair types for reference. The data in this table shows the absolute and relative error from each HE, representing minor differences when comparing it to the acquired spectra. For PETN variations, one variation occurred at 1302 cm^−1^ with a relative error of 0.85%, RDX variation is situated at 1278 cm^−1^ with a relative error of 0.24%, and for TNT, a substantial variation on the loadings plots on 1375 cm^−1^ with a relative error of 0.95%. All these variations are attributed to the -NO2 group in each explosive.

## 3. Discussion

The MVA preliminarily allowed the detection and discrimination of HEs from 300 to 2000 cm^−1^. This chosen range aligns with the vibrational peaks of nitro groups, particularly between 1200 cm^−1^ and 1400 cm^−1^. A PCA model was created for black, bleached, and gray hair as an exploratory tool for the acquired data. However, the raw PCA models did not provide enough information regarding separating the explosives, PETN and RDX, on black and bleached hair. TNT was fully separated on the raw score plot since the vibration of the NO_2_ is from an aromatic compound, and this peak is presented in a relatively wide band.

Since the RS spectra of gray hair samples do not exhibit a strong fluorescence background like the other two hair types, they provided a good separation on their raw score plot. Because the raw score plots were insufficient for representing the spectral data, pretreatments were evaluated. The first and second Savitzky–Golay derivatives yielded the best pre-processing of the results. For black hair, the first derivative applied to the spectral data provides an interval of confidence of 95%. The first derivative could not describe the data after the first principal component for bleached and gray hair, so these datasets were pre-treated with a second derivative. The score plots were analyzed to interpret the variation that could occur from the data.

All the generated models presented a tendency of line clusters, in which the nearer the center, the less the NO_2_ vibrational peak was present on the RS spectra. As it moved away from the center of the plot (position 0,0), the intensities of the prominent peaks for -NO_2_ vibrations tended to increase. Moving away from the center indicated an increase in the intensities of prominent -NO_2_ vibrational peaks, with an additional trend suggesting that -NO_2_ vibration incrementally increased with crystal size. The location of explosive crystals on the hair surface may influence the results, as regions with high fluorescence can obscure the characteristic RS peaks of the HE. The selection of the score plot prompted further examination of variable variation through the provided loadings plot, confirming that the model’s variability was attributed to the distinct nitro groups present in each explosive. However, it is imperative to conduct additional analyses to establish a correlation between crystal position on the hair surface and its contribution to fluorescence in RS spectra.

## 4. Materials and Methods

Hexahydro-1,3,5-trinitro-1,3,5-triazine (RDX) and pentaerythritol tetranitrate (PETN) were synthesized in the laboratory; TNT (min. 30 wt.% water) was obtained from Chem Service (West Chester, Pennsylvania). Women’s and men’s black and gray hair samples were collected from beauty salons and barbershops in Mayaguez, Puerto Rico. Bleached hair was processed from black hair samples at the laboratory. An inVia™ confocal RS microspectrometer (Renishaw, Inc., West Dundee, IL, USA) equipped with a 660 nm diode excitation laser (Cobolt Hubner, Inc., San Jose, CA, USA) was used to detect HEs/hair samples. A total of twenty (20) RS measurements were performed in static mode for each combination of hair type and high explosive (HE), resulting in comprehensive spectral data across all three hair types and three explosives. The acquisition parameters were carefully optimized as follows: acquisition time of 1 second, laser power set to 5% using a neutral density wheel, 100 accumulations per spectrum, and a 50× objective lens. The nominal laser power at the head was 30 mW, resulting in an estimated sample exposure of approximately 1.5 mW. High confocality was maintained throughout the spectral acquisition to enhance spatial resolution and reduce background interference.

Hair samples of ~2 inches were directly exposed to 50 mg of each HE on glass vials and shaken with a mini vortex to promote this adsorption. This approach was considered to obtain preliminary results of the samples to analyze them qualitatively. The hair surface was screened to acquire its spectra on the HE crystals. Further evaluation needs to be addressed with a concentration-based analysis. For further details, see F. Colón-González et al. [23]

The Origin Pro™ 2020 software was used to apply a normalization algorithm to spectral data. Spectral data were processed using WiRE™ CIM 7.6 software (Renishaw Inc.), including baseline correction to remove fluorescence background and normalize the spectra for comparative analysis. MVA for the spectral data was carried out using the Unscrambler-X™ 11.0 software package (CAMO Analytics, Montclair, NJ, USA). After acquiring the data, The Unscrambler-X™ software was used to pre-process the RS spectral data and create multiple models for the data. The PCA models were constructed using the pre-treated data with the first and second Savitzky–Golay derivatives. These derivatives were created using a second-order polynomial fit of 15 points (pts.).

## 5. Conclusions

The exploration of spectroscopic characterization for high explosives (HEs) in human hair remains limited in current research. This study aims to provide a preliminary assessment of the interaction of data from black, bleached, and gray hair on organic surfaces, specifically human hair, by combining RS and PCA. This evaluation enhances our comprehension of their behavior, establishing a foundational reference for future forensic methodologies. The specialized approach resides in screening, monitoring, and mitigation strategies for possible HE threats. Sensor fusion with spectroscopic techniques such as RS, coupled with chemometrics/MVA protocols, can allow fast detection in situ to decrease analysis time, reducing the cognitive load from analysts. This study would enable the extrapolation of the resulting models and libraries for use in other surveillance areas, where in situ and noninvasive techniques are in high demand for citizen protection, personnel security, and other applications.

## Figures and Tables

**Table 1:** Laser beam spot diameter for various ULWD Olympus® microscope objectives: predicted vs. measured.

Objective Magnification	Predicted (μm)	Measured (µm)	f/#
5×	15	12.5	0.77
10×	10	6	0.74
20×	5	3	0.76
50×	3	1.1	0.75
100×	1	0.5	0.70

**Table 2:** Comparison of the experimental vibrational signatures from the PCA models with the RS shift obtained from the loadings plot for black hair.

		HE	HE RS Shift (cm^−1^)	Loadings RS Shift (cm^−1^)	Absolute Error (%)	Relative Error (%)
**PC-1**	**Black Hair**	**PETN**	1291 621	1302 621	11 0	0.85 0
		**RDX**	1275 1218 885	1278 1224 885	3 6 0	0.24 0.49 0
		**TNT**	1618 1534 1362 824	1629 1547 1375 820	11 13 13 4	0.68 0.85 0.95 0.49
**PC-2**	**Black Hair**	**PETN**	1291 624	1283 617	8 7	0.62 1.12
		**RDX**	1310 1275	1323 1283	13 8	0.99 0.63
		**TNT**	1364 824	1375 821	11 3	0.81 0.36

## Data Availability

The data for this study can be found under the corresponding authors’ profiles at https://www.uprm.edu/ccs-cisac/.

## Abbreviations

The following abbreviations are used in this manuscript:

RDX	1,3,5-trinitroperhydro-1,3,5-triazine
TNT	2,4,6-trinitrotoluene
PETN	pentaerythritol tetranitrate
HEs	High Explosives
MVA	Multivariate Analysis
PCA	Principal Component Analysis
RS	Raman Scattering
PC-1	Principal Component 1
PC-2	Principal Component 2
MSC	Multiplicative Scatter Correction
SNV	Standard Normal Variate
BC	Baseline Correction
